# Assessment of the wheat growth-promoting potential of *Delftia lacustris* strain NSC through genomic and physiological characterization

**DOI:** 10.3389/fmicb.2025.1576536

**Published:** 2025-06-16

**Authors:** Pinki Sharma, Rajesh Pandey, Nar Singh Chauhan

**Affiliations:** ^1^Department of Biochemistry, Maharshi Dayanand University, Rohtak, Haryana, India; ^2^INtegrative GENomics of HOst-PathogEn (INGEN-HOPE) Laboratory, CSIR-Institute of Genomics and Integrative Biology (CSIR-IGIB), New Delhi, India; ^3^Academy of Scientific and Innovative Research (AcSIR), Ghaziabad, India

**Keywords:** biocontrol agents, biofertilizer, *Delftia*, food security, rhizosphere microbiota, sustainable agriculture, wheat yield

## Abstract

**Background:**

*Delftia* species have attracted significant interest for their biofertilizer and biocontrol capabilities, particularly in promoting the growth of crops such as *Oryza sativa*, *Brassica campestris*, and *Solanum lycopersicum*. However, their potential in supporting wheat cultivation remains largely unexplored.

**Methods:**

A culture-dependent approach was employed to isolate a *Delftia* strain from the wheat rhizosphere. The biofertilizer potential of the isolate was systematically evaluated through a series of physiological, biochemical, and molecular assays, as well as field trials to assess its efficacy under agronomic conditions.

**Results and discussion:**

Culture-dependent investigation of the wheat rhizosphere led to the isolation of a multifunctional plant growth-promoting bacterium, designated as strain NSC. Morphological and physiological characterization identified NSC as a gram-negative, rod-shaped, motile bacterium with optimal growth at pH 7.0 and 35°C. Phylogenetic and phylogenomic analyses confirmed its taxonomic identity as *Delftia lacustris*. *In vitro* assays revealed its ability to solubilize phosphate (0.325 IU), reduce nitrate (0.401 IU), produce indole-3-acetic acid (0.485 IU), and exhibit ACC deaminase activity (0.512 IU) and siderophore production. The strain demonstrated strong antifungal activity against *Fusarium oxysporum* and *Rhizoctonia solani*. Strain NSC exhibited significant tolerance to abiotic stresses, including drought [up to 40% PEG (w/v)], heavy metals, and high salinity [up to 11.69% NaCl (w/v), 11.18% KCl (w/v), and 4.24% LiCl (w/v)]. Genome analysis identified key genes associated with phosphate solubilization (PhoR, PhoB, PhoU, PstABCD), nitrogen fixation (nifC, nifU), auxin and siderophore biosynthesis, rhizosphere colonization, and antifungal mechanisms (chitinase, PhnZ). In planta studies showed significantly enhanced seed germination (93.33% ± 0.23), seedling growth, and biomass accumulation under stress conditions (*p* < 0.05). Field trials further validated the strain’s efficacy, showing marked improvements in plant growth and yield parameters (*p* = 0.0001). These results underscore the potential of *D. lacustris* NSC as an effective biofertilizer and biocontrol agent for sustainable agriculture.

**Conclusion:**

*Delftia lacustris* strain NSC exhibits multifunctional plant growth-promoting and biocontrol activities, including enhanced nutrient mobilization, pathogen suppression, and abiotic stress tolerance. Its demonstrated efficacy under field conditions and environmentally benign profile highlight its potential as a sustainable bioinoculant for wheat production systems.

## Introduction

The increasing global emphasis on sustainable agriculture has spurred interest in plant growth-promoting rhizobacteria as environmentally friendly alternatives to chemical fertilizers and pesticides (Chandran et al., 2021). Among various genera, *Delftia* has gained attention due to its metabolic versatility (Braña et al., 2016), environmental adaptability (Yin et al., 2022), and plant-associated beneficial traits (Yin et al., 2022). *Delftia* species are motile, aerobic, non-fermentative, gram-negative rods classified under *Betaproteobacteria* (Shigematsu et al., 2003). These bacteria thrive in diverse ecological niches, including wastewater, contaminated soils, clinical samples, and both the rhizosphere and endosphere of plants, highlighting their resilience and functional diversity (Fan et al., 2023). Several *Delftia* strains have demonstrated plant growth-promoting and biocontrol properties.

*Delftia* sp. Lp-1 and *D. acidovorans* DSM 39 possess nitrogen-fixation abilities, allowing them to function as free-living diazotrophs that enhance soil nitrogen levels (Han et al., 2005; Agafonova et al., 2017). *D. tsuruhatensis*, *Delftia* sp. JD2, and *Delftia* sp. BTL-M2, have been reported to colonize the roots of important crops like *Solanum lycopersicum*, *Arabidopsis thaliana*, *Medicago sativa*, and *Pisum sativum* (Morel et al., 2015; Brambilla et al., 2022; Islam et al., 2023). These root-colonizing strains enhance plant development by promoting nutrient availability, synthesizing phytohormones, facilitating root elongation, and lateral root formation (Morel et al., 2016). Field studies have further validated the plant growth-promoting effects of *Delftia* strains. Notably, *Paraburkholderia fungorum* BRRh-4 and *Delftia* sp. BTL-M2 improved growth and yield in *Oryza sativa*, highlighting their agricultural potential (Islam et al., 2023). Similarly, *D. tsuruhatensis* promotes rice growth under nitrogen-deficient conditions by enhancing nutrient solubilization and stimulating root system architecture (Harahap et al., 2023). These strains are also known to produce siderophores, iron-chelating compounds that facilitate iron uptake while restricting iron availability to plant pathogens, thereby serving as indirect biocontrol agents (Guo et al., 2016).

Beyond growth promotion, *Delftia* species are increasingly recognized for their stress-mitigation capabilities (Pattnaik et al., 2021). Many strains exhibit resilience to extreme environmental conditions, particularly heavy metal contamination (Sazykin et al., 2023). *D. lacustris* MS3 has been isolated from lead- and chromium-polluted soils, while *D. acidovorans* SPH-1 mitigates cadmium, zinc, and lead toxicity (Bautista-Hernandez et al., 2012; Samimi and Shahriari-Moghadam, 2021). *D. lacustris* and *Delftia* sp. JD2, have demonstrated resistance to copper, nickel, and hexavalent chromium, making them promising candidates for bioremediation applications (Braña et al., 2016; Morel et al., 2016). Given their well-documented plant growth-promotion, biocontrol activities, and stress tolerance, *Delftia* species hold significant promise as multifunctional bioinoculants. However, most research has focused on horticultural and model crops, such as tomato, rice, and *Arabidopsis*, with limited investigation into their role in cereal crops, particularly wheat.

Wheat is a globally cultivated staple, and enhancing its productivity in a sustainable manner is critical for food security. Modern agricultural practices based on excessive agrochemical use have led to soil degradation, nutrient imbalances, and increased disease susceptibility, reinforcing the need for biological alternatives (Sharma et al., 2024b). The rhizosphere of wheat plants represents a rich microbial niche with untapped potential for discovering novel PGP strains (Sharma et al., 2024a). Given the metabolic traits observed in other *Delftia* isolates (Islam et al., 2023), it is believable that wheat-associated *Delftia* strains may possess similar or enhanced biofertilizer and biocontrol attributes. However, these need to be experimentally validated. Hereby, the present study was planned to assess the biofertilizer and biocontrol properties of the wheat rhizospheric *Delftia* isolate. The impact of *Delftia* isolate needs to be checked on wheat seed germination in the presence of phytopathogens and saline conditions, followed by its impact on plant growth and yield to confirm biofertilizer and biocontrol properties. Moreover, *Delftia’s* ability to tolerate abiotic stresses makes it an ideal candidate for deployment in marginal or degraded agricultural lands. However, the stress response physiology of the wheat rhizosphere *Delftia* isolate needs to be checked through experimentation. In this context, the present study was designed to comprehensively characterize the isolate through physiological, biochemical, and genomic analyses. The central objective was to validate *the Delftia* strain’s potential to promote wheat growth, enhance nutrient uptake, and improve resilience to both biotic and abiotic stresses. Ultimately, these observations would confirm the biocontrol and biofertilizer potential of wheat rhizospheric *Delftia* isolate before its employment to sustainably enhance wheat crop yield.

## Materials and methods

### Study design

**Figure 1:**
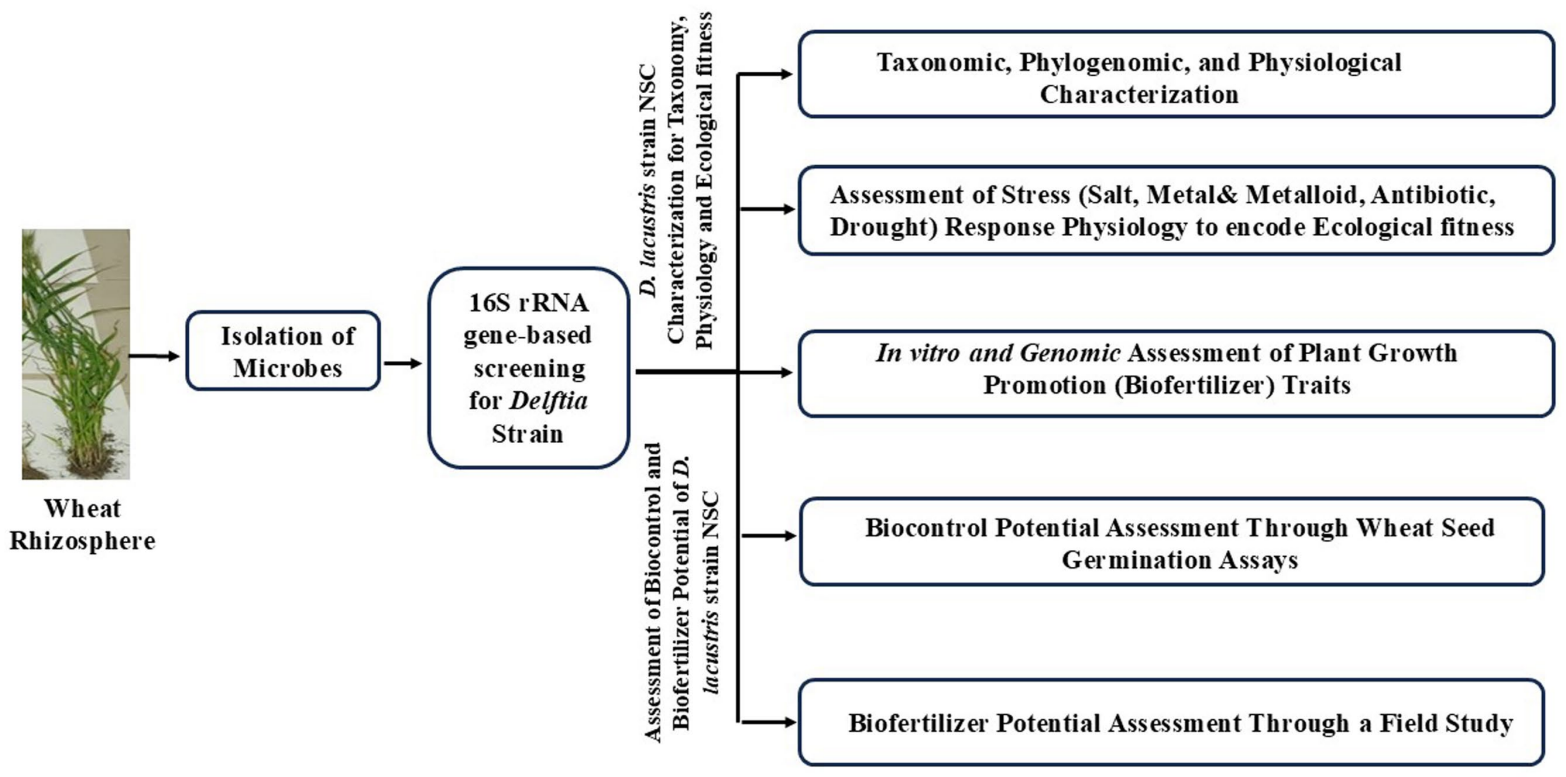
Study design showing the adopted workflow to achieve the proposed objectives.

The present study was designed to assess the ecological fitness, biofertilizer, and biocontrol potential of wheat rhizospheric *D. lacustris* strain NSC. A workflow was developed to assess these characteristics of *D. lacustris* strain NSC (Figure 1).

### Isolation of wheat rhizosphere microbes (WRMs)

Wheat rhizospheric microbes were isolated using a previously standardized method (Supplementary Material 1) (Sharma et al., 2024a). Briefly, rhizospheric soil was collected from randomly selected wheat plants (05) cultivated in an experimental field at Maharshi Dayanand University, Rohtak (28° 52′44” N, 76° 37′19″E), Haryana, India. Wheat rhizosphere samples were harvested with a sterile scoop and immediately transferred to a sterile bag, which was transported immediately to the laboratory for isolation of microbes. Roots were mechanically shaken in the sterile bag to collect the rhizospheric soil. Rhizospheric soil was transferred aseptically in a sterile tube for isolation of rhizospheric microbes following standardized methodology (Supplementary Material 1) (Sharma et al., 2024a). Phylogenetic affiliation of the isolated microbial strains was assessed through 16S rRNA gene-based phylotype identification (Sharma et al., 2024b).

### Molecular, physiological, biochemical, and genomic characterization of *Delftia* strain NSC

Gram staining was performed with a Gram staining kit (K001-1KT, Himedia). The bacterial growth was observed at different pH ranges (3, 4, 5, 7, 8, 9, 10, 11, and 12) and temperature ranges (10°C, 20°C, 30°C, 40°C, 50°C, 60°C) to identify its optimal growth conditions. *Delftia* strain NSC growth pattern was observed in LB broth for 48 h at 37°C with constant shaking at 200 rpm to check its growth pattern and generation time (Supplementary Material 2) (Sharma et al., 2024a). Substrate utilization preference was assessed with a Hi-Carbo kit (Himedia, KB009A-1KT, KB009B-1KT, and KB009C-1KT) (Supplementary Material 3). Biochemical properties were performed using assays for amylase (Swain et al., 2006), catalase (Iwase et al., 2013), pectinase (Oumer and Abate, 2018), cellulase (Kasana et al., 2008), esterase (Ramnath et al., 2017), and protease (Vijayaraghavan et al., 2017) (Supplementary Materials 4–9). The stress response physiology was assessed by performing specific assays for salt stress tolerance, metal stress tolerance, and oxidative stress (Supplementary Material 10) (Sharma et al., 2024a). DNA was extracted from the microbial isolate using the alkali lysis method (Chauhan et al., 2009). The qualitative and quantitative analysis of the DNA was performed with agarose gel electrophoresis and Qubit HS DNA estimation kits (Invitrogen, United States), respectively. Genomic DNA was sequenced using Illumina MiSeq platform using Nextera XT DNA Library Prep kit (Yadav et al., 2022) to unveil its genomic architecture. Quality filtration, genome assembly, and genome completeness were checked using standard methodology (Yadav et al., 2022). Genomic characterization and comparative genomics were performed against other *Delftia* using a standardized methodology (Supplementary Material 11) (Yadav et al., 2022).

### Assessment of biofertilizer-related features in *D. lacustris* strain NSC

*Delftia lacustris* strain NSC was characterized for its biofertilization potential through a series of functional assays, including phosphate solubilization (Behera et al., 2017), nitrate reductase activity (Kim and Seo, 2018), indole-3-acetic acid (IAA) production (Ehmann, 1977), ammonia production (Bhattacharyya et al., 2020), and siderophore biosynthesis (Himpsl and Mobley, 2019) (Supplementary Materials 2–16). Its drought stress tolerance property was also evaluated (Supplementary Material 17) (Mustamu et al., 2023). Its efficiency to mitigate salt-induced oxidative stress was evaluated by measuring its 1-aminocyclopropane-1-carboxylic acid (ACC) deaminase activity (Maheshwari et al., 2020) (Supplementary Material 18). The RAST software assessed the genetic features related to plant growth promotion.^1^

### Assessment of *Delftia lacustris* strain NSC biofertilizer and biocontrol properties in laboratory conditions

The biocontrol potential was evaluated against *Rhizoctonia solani*, and *Fusarium oxysporum* (Supplementary Material 19). Both of these phytopathogens are the most prevalent and economically damaging soil-borne fungi affecting wheat, known to cause root rot, damping-off, and vascular wilt, which significantly reduce crop yield and quality (Wille et al., 2019; Rhouma et al., 2024). Pre-inoculation of seeds with biofertilizer strains has been known to improve plant growth parameters like shoot and root length, etc. (O'Callaghan, 2016; Kumar et al., 2023). The assessment focused on their effects on seed germination efficiency and wheat seedlings’ root and shoot lengths (Singh and Kayastha, 2014; Sharma et al., 2024a). To investigate the role of *D. lacustris* strain NSC in seed germination under saline conditions, seeds were soaked in an overnight-grown microbial culture (cell density of 10^11^ cells/mL), supplemented with NaCl concentrations ranging from 0 to 1 M, for 16 h at 37°C. Control seeds were soaked directly in NaCl solutions of equivalent concentrations for 16 h at 37°C. Following the soaking treatment, seeds were wrapped in germination sheets and placed in 50 mL culture tubes containing 5 mL of Hoagland solution. The tubes were incubated in the dark at ambient temperature (25°C) for 7 days. After incubation, wheat seed germination percentage, alpha-amylase activity, and root and shoot lengths were measured (Singh and Kayastha, 2014; Sharma et al., 2024a).

### Assessment of *D. lacustris* strain NSC biofertilizer properties under field conditions

Roots of wheat cultivar (WC)-306 plants treated with *D. lacustris* strain NSC were harvested at various Feeks and assessed for their phosphate-solubilizing ability (Behera et al., 2017), nitrate reductase activity (Kim and Seo, 2018), reducing sugar content (Khatri and Chhetri, 2020), and total sugar content (Ludwig and Goldberg, 1956) (Supplementary Materials 20–25). These results were compared to those of untreated WC-306 plants. In addition, several growth and yield parameters were evaluated in both treated and untreated plants, including seed germination percentage, number of tillers per plant, number of leaves per plant, number of spikes per plant, spike length, number of spikelets per spike, and grain yield, to assess the impact of *D. lacustris* strain NSC on wheat growth and productivity (Supplementary Material 26).

### Statistical analysis

All experiments were performed with replicates (a total of six replicates for microbial physiological and biochemical studies, and 10 replicates for all plant-based studies). Statistical analyses and data visualization were conducted using SIGMA Plot 15.0. Significant difference between microbial-treated and control groups for growth parameters (number of tillers, number of leaves per plant, spike length, number of spikes per plant, number of spikelets per plant, grain weight per 1,000 grains, and grain yield was determined using One-Way ANOVA using Systat Software, Sigma Plot 15.0). Data distribution was checked both visually (histogram analysis) and statistically (Shapiro–Wilk Test) before performing ANOVA analysis.

## Results

### Identification of microbial isolate NSC

**Figure 2:**
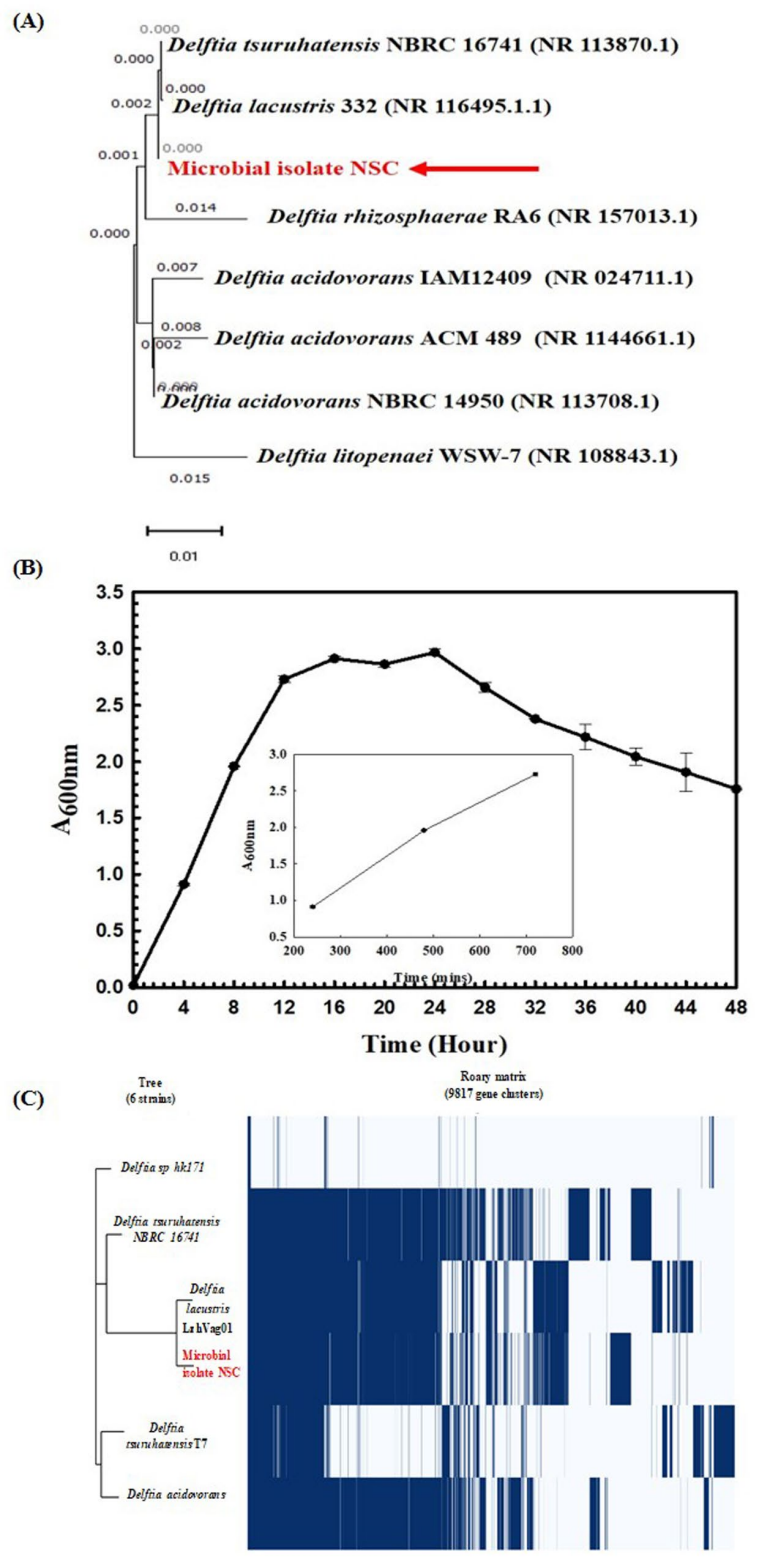
Phylogenetic and Physiological characterization of *Delftia lacustris* strain NSC. Phylogenetic affiliation of microbial isolates NSC with the other *Delftia* species **(A)**. The phylogenetic tree was constructed using the Neighbor-joining method of phylogenetics with 1,000 bootstrap replications using MEGA-X software. The out-group was represented by *Delftia litopenaei* WSW-7 SSU rRNA gene sequence. Growth pattern analysis of *Delftia lacustris* strain NSC was observed after incubating the cultures for 48 h in LB broth with constant shaking at 200 rpm. The inset figure represents the linear growth phase used to calculate generation time **(B)**. Phylogenomic analysis of *D. lacustris* strain NSC with other *Delftia* strains **(C)**. The phylogenomic tree of the microbial isolate was generated using the FastTree v2.1.10 tool through Roary. The left panel illustrates the phylogenetic relationship of *D. lacustris* NSC with other *Delftia* species, while the right panel shows the core and accessory genes shared among different *D. lacustris* strains.

Wheat rhizosphere has a pH of 7.3 ± 0.0025, a temperature of 22.6°C ± 0.01, and a moisture content of 11.5 ± 1.10014. A total of 12 microbial isolates were obtained from the rhizosphere. The 16S rRNA gene-based taxonomic characterization revealed that only NSC isolate’s 16S rRNA gene shared 99.86% homology with *Delftia lacustris* strain 332 (NR 116495) in the NCBI Nr database. This identification was further confirmed through phylogenetic analysis (Figure 2A). Based on this observation, the isolated NSC was used for further characterization.

### Molecular, physiological, biochemical, and genomic characterization of *D. lacustris* strain NSC

Microscopic examination identified the isolate as a Gram-negative, rod-shaped, motile bacterium. Physiologically, it exhibited optimal growth at pH 7.0 and 35°C temperature, reaching the logarithmic phase after 12 h with a generation (doubling) time of approximately 54.31 min (Figure 2B). Growth profile analysis at various temperatures and pH conditions indicated its sustained growth between 10°C and 50°C and pH 5 and 8. It also demonstrated facultative anaerobic growth, with an optical density (O. D.) of 0.402 at 600 nm after 24 h under anaerobic conditions. Biochemical testing showed it was positive for amylase, esterase, lipase, protease, and catalase activities, and its substrate utilization profile was consistent with that of other *Delftia* species (Supplementary Table S1). Antibiotic susceptibility testing revealed that it was resistant to amoxicillin, bacitracin, cephalothin, vancomycin, ceftazidime, and ofloxacin, but remained sensitive to oxytetracycline, novobiocin, and lincomycin, resembling the antibiotic resistance profile of other *Delftia* strains (Supplementary Table S2).

Stress response assays demonstrated that it could tolerate saltine stress [up to 11.69% NaCl (w/v), 11.18% KCl (w/v), 4.24% LiCl (w/v)], metal stress [0.068% CdCl_2_ (w/v), up to 0.1% Na_3_AsO_4_ (w/v), 0.15% NaAsO_2_ (w/v)], and oxidizing agents stress [up to 4.25% H_2_O_2_ (v/v)]. These properties were found similar to other *Delftia* strains (Supplementary Table S3). These findings highlight the adaptability of the *D. lacustris* strain NSC under stress conditions. Genome sequencing has generated 805,929 paired-end raw reads, which were assembled into 6,235,469 base pairs represented through 220 contigs. Functional annotation of the genome revealed 5,466 protein-coding sequences, 8 rRNA genes, 82 tRNA genes, and one tmRNA gene. The Average Nucleotide Identity (ANI) among different species of *Delftia* sp. ranged from 74 to 98%, reflecting significant genomic variation between species. Notably, the ANI between *D. lacustris* NSC and *Delftia lacustris* LzhVag01 was 98.28%, higher than those observed with other species within the genus (Supplementary Table S4). The classification of *D. lacustris* strain NSC as part of the *Delftia* species was further confirmed using tetra correlation. *Delftia* sp. LMG 24775 showed a z-score of 0.99989 when compared to *D. lacustris* strain NSC, confirming its close genetic relationship. Other *Delftia* species exhibited high similarity, with z-scores ranging from approximately 0.95–0.99 (Supplementary Table S5). Following ANIb and tetra confirmation, the genome of *D. lacustris* strain NSC was compared to those of other *Delftia* species to assess genome-wide similarities and differences. A matrix generated using the Roary tool highlighted the extensive nature of the genome, showing that *D. lacustris* strain NSC exhibited the greatest similarity to *D. lacustris* LzhVag01 (Figure 2C). The Roary analysis also revealed that all *Delftia* genomes shared a significant number of genes. Notably, the *D. lacustris* strain NSC genome lacks pathogenic genes or virulence-related genes, supporting its non-pathogenic nature.

Genomic analysis also identified genes involved in phosphate solubilization and transport (Table 1). Beyond phosphate solubilization, its genome includes genes associated with plant growth promotion activities, such as auxin biosynthesis, nitrogen assimilation, and siderophore biosynthesis (Table 1). Presence of genes for arsenic resistance, oxidative stress tolerance, metal stress tolerance, and salt tolerance, confirming the genome-derived stress-mitigating physiology of *D. lacustris* strain NSC. A detailed examination of the *D. lacustris* strain NSC genomes also revealed genes encoding proteins for the synthesis of Type 1 and IV pili and exopolysaccharides (Table 1), crucial for plant surface adhesion, auto-aggregation, and early biofilm formation.

**Table 1:** Genetic features identified within *Delftia lacustris* strain NSC genome encoding various proteins involved in nutrient assimilation, solubilization, colonization, and antifungal properties.

Start	Stop	DNA Strand*	Function
Auxin biosynthesis			
45,707	49,288	+	Indolepyruvate ferredoxin oxidoreductase, alpha and beta subunits
34,647	33,844	−	Indole-3-glycerol phosphate synthase (EC 4.1.1.48)
59,394	58,441	−	Auxin efflux carrier family protein
36,653	37,603	+	Auxin efflux carrier family protein
Nitrogen metabolism, assimilation, transport and fixation			
56,709	55,972	−	Respiratory nitrate reductase gamma chain (EC 1.7.99.4)
57,419	56,706	−	Respiratory nitrate reductase delta chain (EC 1.7.99.4)
58,972	57,431	−	Respiratory nitrate reductase beta chain (EC 1.7.99.4)
62,845	58,991	−	Respiratory nitrate reductase alpha chain (EC 1.7.99.4)
64,373	62,913	−	Nitrate/nitrite transporter NarK/U
65,712	64,390	−	Nitrate/nitrite transporter NarK/U 1
71,965	71,294	−	Nitrate/nitrite response regulator protein
73,892	71,994	−	Nitrate/nitrite sensor protein
3	413	+	Nitrite reductase [NAD(P)H] large subunit (EC 1.7.1.4)
43,943	43,602	−	Nitrite reductase [NAD(P)H] small subunit (EC 1.7.1.4)
46,492	43,976	−	Nitrite reductase [NAD(P)H] large subunit (EC 1.7.1.4)
20,232	20,684	+	Nitrite-sensitive transcriptional repressor NsrR
6,488	7,753	+	Response regulator NasT
9,332	9,613	+	Nitrogen-fixing NifU, C-terminal
Phosphate solubilization, regulation and transport			
155,008	154,274	−	Phosphate transport system regulatory protein PhoU
155,799	155,026	−	Phosphate ABC transporter, ATP-binding protein PstB
156,734	155,844	−	Phosphate ABC transporter, permease protein PstA
157,693	156,731	−	Phosphate ABC transporter, permease protein PstC
158,824	157,781	−	Phosphate ABC transporter, substrate-binding protein PstS
−	−	−	Phosphate ABC transporter, substrate-binding protein PstS
103,243	103,932	+	Phosphate regulon sensor protein PhoR (SphS)
103,947	105,260	+	Phosphate regulon transcriptional regulatory protein PhoB (SphR)
Siderophore biosynthesis			
44,790	45,422	+	Ferric siderophore transport system, biopolymer transport protein ExbB
51,889	54,375	+	TonB-dependent siderophore receptor
98,521	96,089	−	Ferric siderophore receptor, TonB dependent
43,035	41,314	−	ABC-type siderophore export system, fused ATPase and permease components
51,149	48,750	−	Outer membrane ferripyoverdine receptor FpvA, TonB-dependent @ Outer membrane receptor for ferric siderophore
69,785	51,333	−	Siderophore biosynthesis non-ribosomal peptide synthetase modules
79,804	69,857	−	Siderophore biosynthesis non-ribosomal peptide synthetase modules
54,507	56,435	+	TonB-dependent siderophore receptor
74,393	71,766	−	TonB-dependent siderophore receptor
12,835	15,351	+	TonB-dependent siderophore receptor
6,504	5,542	−	Ferric siderophore ABC transporter, permease protein
18,584	16,110	−	Ferric siderophore receptor, TonB dependent
105,054	107,222	+	TonB-dependent siderophore receptor
85,709	83,583	−	Outer membrane (iron. B12.siderophore.hemin) receptor
Colonization (Pili formation Protein)			
84,316	85,008	+	Minor pilin of type IV secretion complex, VirB5
85,244	86,164	+	Inner membrane protein of type IV secretion of T-DNA complex, VirB6
86,199	86,483	+	Lipoprotein of type IV secretion complex that spans outer membrane and periplasm, VirB7
86,488	87,159	+	Inner membrane protein forms channel for type IV secretion of T-DNA complex, VirB8
87,156	88,013	+	Outer membrane and periplasm component of type IV secretion of T-DNA complex, has secretin-like domain, VirB9
88,025	89,197	+	Inner membrane protein of type IV secretion of T-DNA complex, TonB-like, VirB10
89,206	90,225	+	ATPase required for both assembly of type IV secretion complex and secretion of T-DNA complex, VirB11
37,606	36,884	−	Minor pilin of type IV secretion complex, VirB5
Mannose-6-phosphate isomerase			
624,120	625,055	+	Mannose-6-phosphate isomerase (EC 5.3.1.8)
22,281	23,219	+	Mannose-6-phosphate isomerase, class I (EC 5.3.1.8)
EPS biosynthesis			
7,311	6,520	−	Exopolysaccharide production negative regulator ExoR
34,776	35,345	+	Transcriptional activator of exopolysaccharide II synthesis, MarR family protein
59,044	57,788	−	exopolysaccharide production protein, putative
64,636	66,402	+	Uncharacterized protein involved in exopolysaccharide biosynthesis
33,244	34,539	+	Exopolysaccharide production protein ExoF precursor

Biocontrol activity			
1965	1,210	−	Intracellular protease
4,618	3,428	−	trypsin-like serine protease
10,019	8,076	−	Extracellular protease precursor (EC 3.4.21.-)
167,991	170,144	+	Protease II (EC 3.4.21.83)
31,048	33,105	+	putative ATP-dependent protease
31,604	33,109	+	Serine protease
13,947	15,482	+	Probable exported protease
62,237	62,791	+	Intracellular protease
17,501	18,304	+	Phenazine biosynthesis protein PhzF like
34,805	35,764	+	Phenazine biosynthesis protein PhzF like

*Here, + and – represent leading and lagging DNA strands.

### Assessment of biofertilizer-related features in *D. lacustris* strain NSC

*Delftia lacustris* strain NSC demonstrated phosphate solubilizing activity (0.325 IU), nitrate reductase activity (0.401 IU), and produced IAA (0.485 IU). The qualitative analysis showed that *D. lacustris* strain NSC could also generate ammonia. Siderophore biosynthesis assays revealed the formation of an orange color and the development of a clearance zone (19.5 ± 0.02 mm), indicating the siderophore production ability. It has also demonstrated strong growth even in the presence of up to 40% polyethylene glycol (PEG). This result suggests that *D. lacustris* strain NSC possesses traits that can help plants withstand drought by enhancing growth in challenging environments. Additionally, it was found to produce the enzyme ACC deaminase, with an activity level of 0.512 IU. ACC deaminase activity suggests that it may play a crucial role in promoting plant health under stressful conditions, such as drought, by reducing oxidative stress and supporting plant growth.

### Assessment of *D. lacustris* strain NSC biofertilizer and biocontrol properties in laboratory conditions

**Figure 3:**
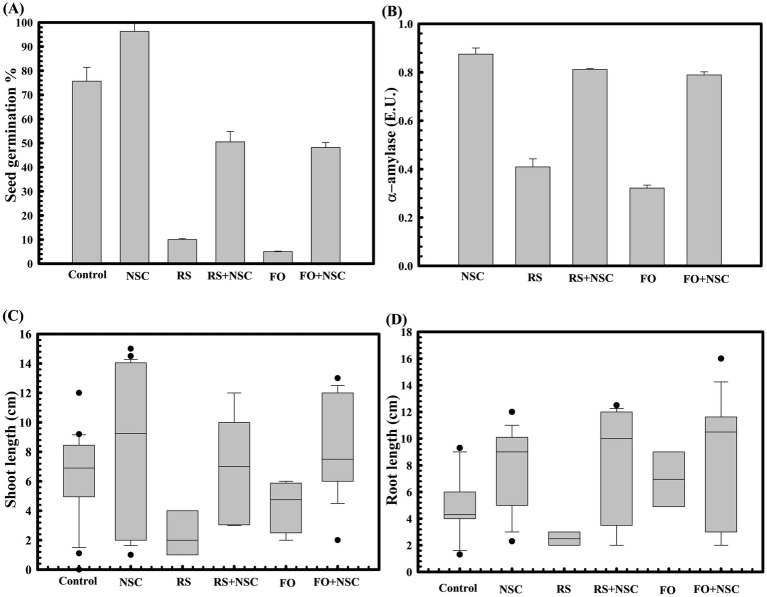
Effect of *D. lacustris* strain NSC on wheat seed germination parameters. The seed germination percentage in *D. lacustris* strain NSC pretreated seed in reference to the control (untreated) **(A)**. The alpha amylase activity of *D. lacustris* strain NSC pretreated wheat seeds in the presence and absence of plant pathogenic fungi *Rhizoctonia solani* and *Fusarium oxysporum* **(B)**. The impact of seeds pretreatment with *D. lacustris* strain NSC on shoot **(C)** and root length **(D)** of WC-306 seedling in the presence of *R. solani* and *F. oxysporum*. Plotted values are the mean of triplicates along with the observed standard deviation. Here NSC, *D. lacustris* NSC; FO, *Fusarium oxysporum*; RS, *Rhizoctonia solani*.

Plant growth promotion properties *of D. lacustris strain NSC were sequentially assessed at different plant growth stages*, starting from seed germination to plant grain yield. Wheat seed germination assay reflected that the untreated control group had a seed germination rate of 75.66 ± 0.57735%. In contrast, seeds pre-treated with *D. lacustris* strain NSC exhibited a germination efficiency of 93.33 ± 0.23% (Figure 3A), reflecting approximately a 123.35-fold increase (*p = 0.0001*) in germination percentage.

The protective effect of *D. lacustris* strain NSC was also assessed against phytopathogen infestation during wheat seed germination. In the presence of *Rhizoctonia solani* and *Fusarium oxysporum*, seed germination was substantially reduced in untreated seeds to 10 ± 1% (*p = 0.024*) and 5 ± 0.57735% (*p = 0.032*), respectively. However, pre-treatment with *D. lacustris* strain NSC led to improved germination efficiencies of 52.25 ± 0.23% and 48.19 ± 0.55% in the presence of *R. solani* and *F. oxysporum*, respectively (Figure 3A). Notably, it has significantly enhanced seed germination in the presence of these phytopathogens by approximately 520-fold (*p = 0.0001*) and 963.8-fold (*p = 0.0001*) compared to untreated control seeds. These findings strongly suggest the biocontrol potential of the *D. lacustris* strain NSC, as it improved seed germination in the presence of phytopathogens and significantly outperformed untreated seeds (*p = 0.0025*) (Figure 3). Moreover, *R. solani* and *F. oxysporum* exposure during wheat seed germination significantly reduced alpha-amylase activity to 0.42 IU and 0.22 IU, respectively. Pre-treatment with *D. lacustris* strain NSC significantly restored alpha-amylase activity to 0.792 IU (*p = 0.0001*) and 0.735 IU (*p = 0.0001*) in the presence of *R. solani* and *F. oxysporum*, respectively (Figure 3B).

Accordingly, the impact of seeds inoculation with strain NSC on plantlet growth parameters was also assessed. Pre-treatment with *D. lacustris* strain NSC also increased wheat plantlets’ shoot and root length. Seeds treated with *D. lacustris* NSC exhibited significantly increased shoot length (*p = 0.033*) (Figure 3C) and root length (*p = 0.002*) (Figure 3D) relative to untreated seeds. Furthermore, it has significantly improved the root (*p = 0.020*, *p = 0.032*) and shoot length (*p = 0.031*, *p = 0.129*) of plantlets derived from wheat seeds exposed to *R. solani* and *F. oxysporum*, respectively. Even after exposure to these pathogens, the average shoot and root lengths of plantlets treated with *D. lacustris* isolate NSC remained significantly higher (*p = 0.0297* and *p = 0.010*) compared to the untreated control, highlighting its biocontrol potential.

**Figure 4:**
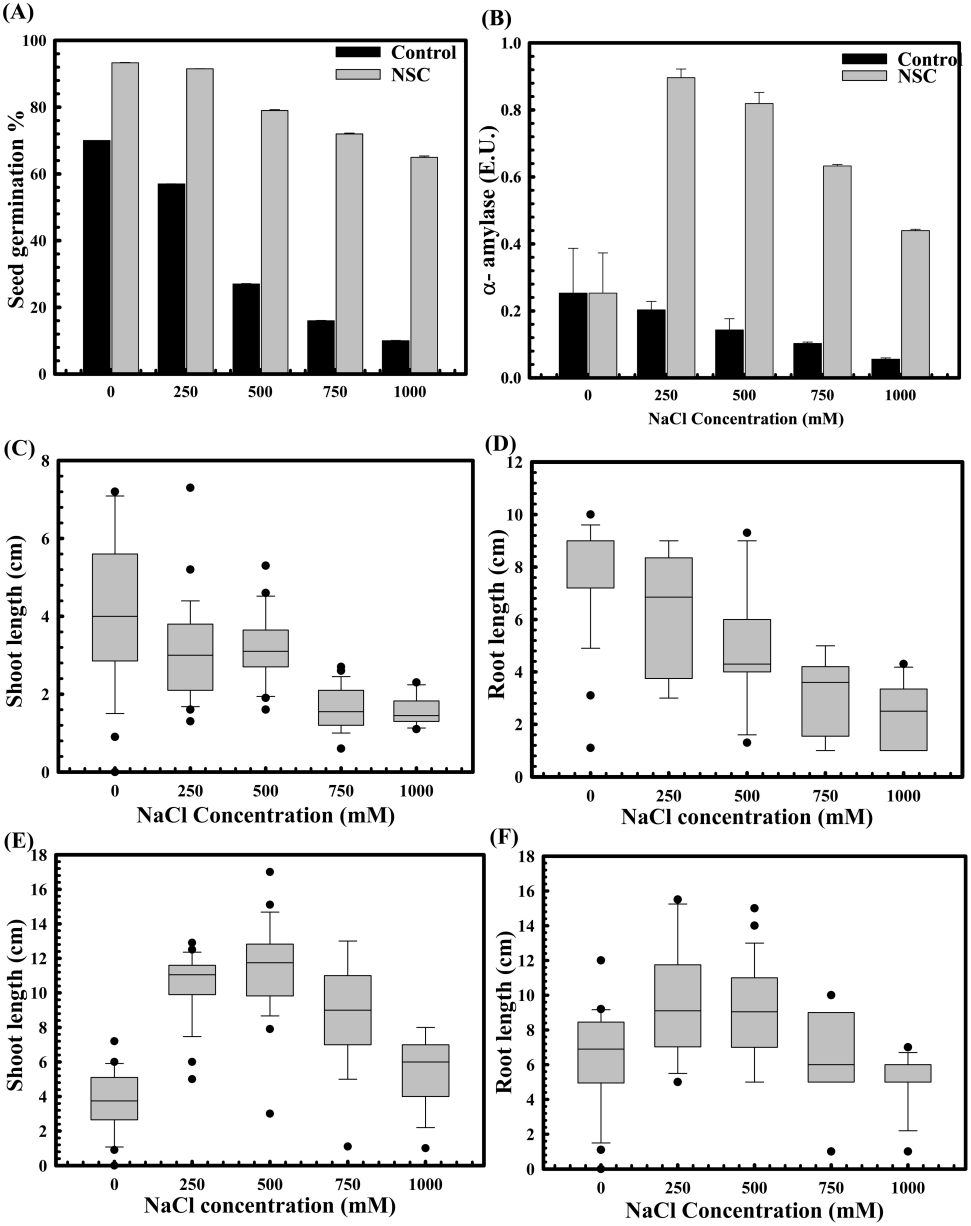
The impact of *D. lacustris* strain NSC pre-treatment on wheat seed germination in saline conditions. The seed germination assays were performed to compare the effect of *D. lacustris* strain NSC on seed germination under saline conditions **(A)**. The alpha amylase activity of *D. lacustris* strain NSC pretreated wheat seeds under saline stress conditions **(B)**. Shoot and root lengths of WC-306 seedlings were assessed under both untreated **(C,D)** and *D. lacustris* strain NSC pre-treated group **(E,F)** under NaCl-induced salinity stress. All assays were performed in triplicate.

The role of *D. lacustris* strain NSC in enhancing wheat seed germination under abiotic stress, such as high salinity, was also evaluated. Germination rates significantly decreased with increasing salt concentrations, up to 500 mM NaCl (Figure 4). However, seeds pre-treated with *D. lacustris* strain NSC exhibited improved germination under high salinity conditions (Figure 4A). Specifically, seed germination increased by approximately 10.11-fold (*p = 0.0001*) at 1 M NaCl, with corresponding increases in alpha-amylase activity (0.412 IU at 1 M NaCl) (Figure 4B) compared to controls. Pre-treatment with it enhanced seed germination and promoted wheat plantlet growth. At the highest NaCl concentration (1 M), seed pre-treatment exhibited a ~4.02-fold increase in shoot length (*p = 0.0003*) (Figure 4E) and a ~2.34-fold increase in root length (*p = 0.0001*) (Figure 4F) compared to untreated seeds under saline conditions (Figures 4C,D).

### Assessment of *D. lacustris* isolate NSC biofertilizer properties under field conditions

**Figure 5:**
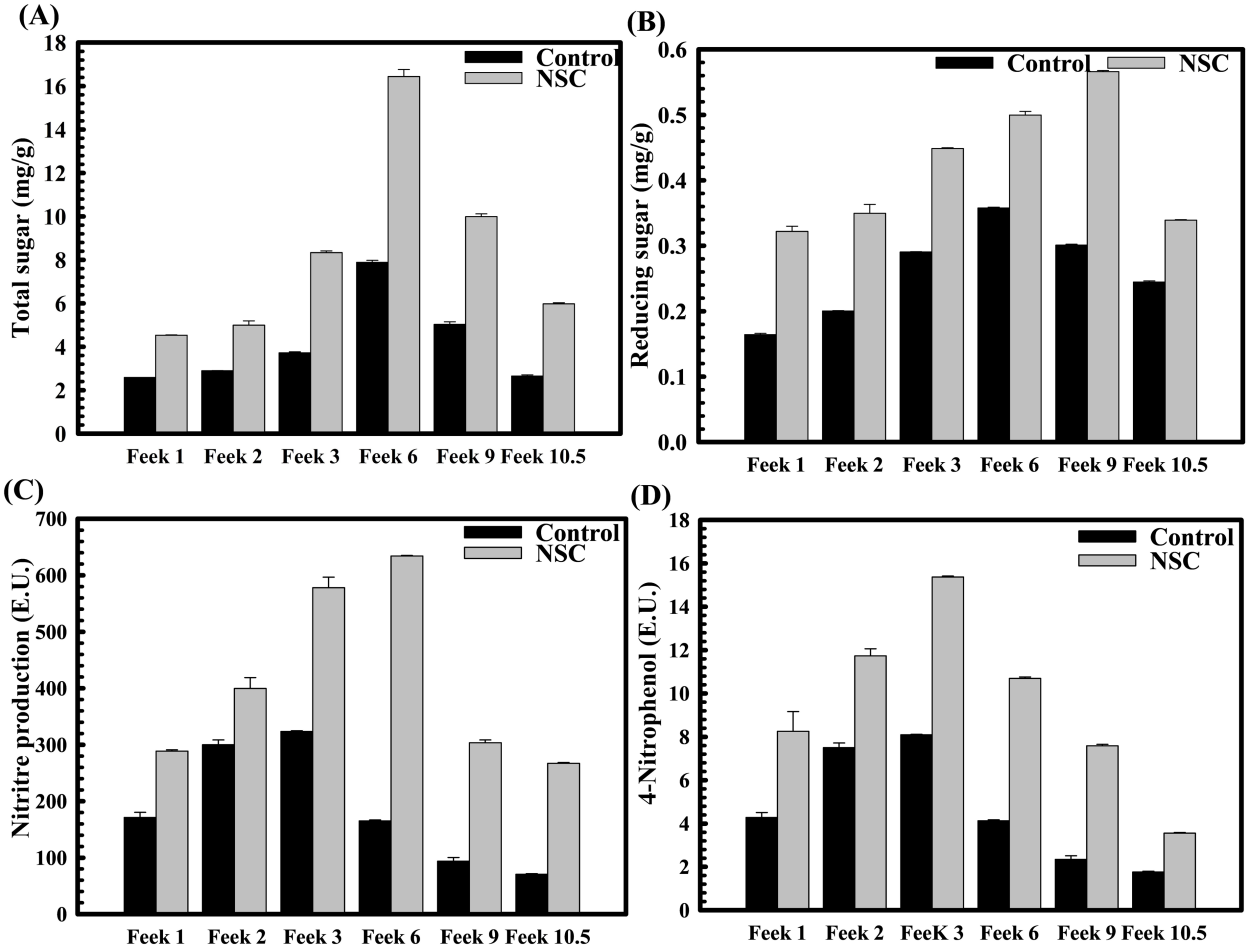
The impact of *D. lacustris* strain NSC pre-treatment on rhizosphere sugar content, phosphatase, and nitrate reduction activity across various wheat growth stages. Total sugar content was observed at different Feeks (1.0, 2.0, 3.0, 6.0, 9.0, and 10.5) in the presence and the absence of microbial inoculant NSC **(A)**. Reducing sugar was estimated using DNS assay at different Feeks (1.0, 2.0, 3.0, 6.0, 9.0, and 10.5) was assessed in the presence and the absence of microbial inoculant NSC **(B)**. Assessment of nitrate reductase activity at different Feeks (1.0, 2.0, 3.0, 6.0, 9.0, and 10.5) in the presence or absence of *D. lacustris* strain NSC **(C)**. Assessment of alkaline phosphatase activity at different Feeks (1.0, 2.0, 3.0, 6.0, 9.0, 10.5) in the presence or absence of *D. lacustris* strain NSC **(D)**. The experiment was carried out in triplicate. Plotted values are the mean of triplicate readings and their observed standard deviation.

The physiological parameters were assessed at wheat plant growth stages, also known as Feeks. The rhizosphere of wheat plants derived from seeds pre-treated with *D. lacustris* strain NSC exhibited substantial enhancements in various physiological parameters such as phosphate solubilization (*p = 0.0025*), nitrogen assimilation (*p = 0.0005*), the release of total sugar (*p = 0.0022*), and reducing sugar (*p = 0.0011*) relative to untreated controls. Across multiple growth stages (Feeks 1, 2, 3, 6, 9, and 10.5), the concentrations of total sugars and reducing sugars in the rhizosphere were significantly elevated in the pre-treated plants, with total sugars increasing by ~2.1 fold increase (Figure 5A), and reducing sugars by ~2.03 fold (Figure 5B). Likewise, extracellular alkaline phosphatase activity was markedly higher in the pre-treated plants, showing increases ranging from ~ 3.48-fold at different stages (Figure 5C). Additionally, pre-treatment resulted in enhanced nitrogen assimilation in the rhizosphere, as evidenced by a substantial increase in nitrate reductase activity. This activity was elevated by ~2-fold at Feeks 3, with consistent increases observed at other stages, ranging from ~6.01-fold higher than that in untreated seeds (Figure 5D). These findings indicate that the pre-treatment facilitated improved nutrient cycling, particularly in relation to sugars and phosphorus, and promoted greater nitrogen uptake, thereby supporting enhanced growth and development of the wheat plants. Wheat plants originated from seeds treated with *D. lacustris* strain NSC showed a significant increase in the number of tillers (*p = 0.0042*), number of leaves per plant (*p = 0.0002*), spike length (*p = 0.0025*), number of spikes per plant (*p* = 0.0033), number of spikelets per plant (*p = 0.0001*), grain weight per 1,000 grains (*p = 0.0011*) and grain yield (*p = 0.0001*) (Table 2). These findings suggested that *D. lacustris* strain NSC enhanced wheat productivity by promoting both vegetative growth (e.g., more tillers and leaves) and reproductive success (e.g., longer spikes, more spikelets, and higher grain weight), leading to higher overall grain yields. This could have important implications for sustainable agriculture, particularly as these microbial isolates may reduce the need for chemical fertilizers and enhance crop performance under varying environmental conditions.

**Table 2:** Assessment of plant productivity features in *D. lacustris* strain NSC treated seeds in comparison to untreated.

Productivity phenotype	WC-306 Plants	WC-306 plants treated with *Delftia lacustris* NSC
Leaves per plant	12.34 ± 0.57	46 ± 0.05
Number of tillers	3.34 ± 0.57	5.53 ± 0.22
Number of spike/plant	26.3 ± 1.309	49.9 ± 1.2
Spike length (cm)	15.46 ± 0.41	22.69 ± 0.52
Number of spikelets per plant	30.34 ± 0.57	59.22 ± 1.02
Grain weight (g)	30.23 ± 0.37	58 ± 0.57
Grain yield (Kg/acre)	3,267	56328.5

All the experiments were performed in triplicate. Values in the table represent the mean and S. D.

## Discussion

In the present study, a bacterial isolate (designated NSC) was obtained from the wheat rhizosphere, a microecological niche known for harboring diverse beneficial microbes (Han et al., 2005; Ubalde et al., 2012). Each microbial isolate must be taxonomically and phylogenetically characterized prior to its employment for any applications (Keswani et al., 2019). Taxonomic and phylogenetic analyses confirmed the affiliation of isolate NSC with *D. lacustris* strain 332. This identification was further supported by phylogenetic clustering and genome-wide similarity metrics, including average nucleotide identity (ANI) and tetra correlation values. Microscopic examination revealed that the isolate is a Gram-negative, rod-shaped, and motile bacterium, consistent with the established morphology of *Delftia* spp. (Grady et al., 2016). All these results ensured the isolation of the target bacterium, i.e., *D. lacustris*, from the wheat rhizosphere. Physiological assessments showed that isolated NSC grows optimally at neutral pH and under mesophilic conditions, but it also tolerates a wide range of temperatures and pH levels. Given that soil is a dynamic ecosystem with fluctuating environmental parameters (Mehta et al., 2021; Morigasaki et al., 2024), microbial adaptability to such changes is crucial. The ability of *D. lacustris* NSC to thrive under varying conditions highlights its ecological fitness and survivability in the soil environment.

Biochemical assays demonstrated the production of several hydrolytic enzymes, including amylase, lipase, esterase, and protease, which play roles in nutrient mobilization and pathogen suppression (Figueiredo et al., 2021). In nutrient-poor soil environments, a microbe’s metabolic versatility supports its survival and functional performance (Hopkins and Dungait, 2010; Wang et al., 2015). Isolate NSC exhibited a broad substrate utilization profile and robust metabolic machinery, positioning it as a strong candidate for soil application as a plant growth-promoting bacterium (PGPB).

Soils are increasingly contaminated by anthropogenic pollutants, particularly heavy metals and metalloids such as cadmium and arsenic (Vareda et al., 2019). These contaminants disrupt soil ecology and impair plant growth (Hou et al., 2025). *Delftia* strains are known for their ability to tolerate salinity and metal/metalloid stress, making them suitable for application in degraded soils (Bautista-Hernandez et al., 2012; Samimi and Shahriari-Moghadam, 2021). In line with this, isolate NSC demonstrated tolerance to multiple abiotic stresses, including salinity, cadmium, arsenic, and oxidative stress (H₂O₂), confirming its environmental resilience. These traits are consistent with previous reports of stress-tolerant *Delftia* strains from contaminated environments (Gulati et al., 2020; Kumar S. et al., 2022; Kumar M. et al., 2022).

Antibiotic accumulation in soil, resulting from manure, pharmaceuticals, sludge, or native microbial activity, poses additional ecological stress (Cycoń et al., 2019; Hashmi et al., 2017). Isolate NSC displayed resistance to multiple antibiotics, including amoxicillin, bacitracin, cephalothin, vancomycin, ceftazidime, netillin, and ofloxacin. Genome-wide analysis revealed the presence of antibiotic resistance genes, suggesting the isolate’s ability to survive in antibiotic-contaminated environments. The combination of an extended substrate range, metabolic richness, and stress tolerance underscores its rhizosphere fitness and colonization potential—key features for an effective biofertilizer (Compant et al., 2005).

This study primarily aimed to evaluate the biofertilizer and biocontrol potential of *D. lacustris* NSC through genomic, physiological, and field-based investigations. Genome analysis revealed numerous plant growth-promoting genes, including those involved in phosphate solubilization, nitrogen assimilation, indole-3-acetic acid (IAA) biosynthesis, siderophore production, and ACC deaminase activity—well-established PGP mechanisms (Glick, 2014; Egamberdieva et al., 2017; Sharma et al., 2024a). The genome also encodes genes for chitinase, proteases, phenazine, and siderophores—critical components for biocontrol via fungal cell wall degradation and iron sequestration (Nefzi et al., 2019; Karmegham et al., 2020). ACC deaminase activity helps mitigate ethylene-induced stress (Danhorn and Fuqua, 2007), while genes for exopolysaccharide biosynthesis and Type I/IV pili support root adhesion, biofilm formation, and rhizosphere competence (Zhang et al., 2024). While concerns exist about the pathogenic potential of some *Delftia* strains (Berg et al., 2005), genomic analysis of *D. lacustris* NSC revealed the absence of known virulence factors, suggesting it is safe for agricultural use.

Phosphate solubilizing activity, nitrate reductase activity, ammonia production, and IAA biosynthesis are a few essential properties of potential biofertilizer strains (Tang et al., 2020). Results have showcased that *D. lacustris* strain NSC harbors all these biofertilizer traits. It was also found to tolerate osmotic stress induced by polyethylene glycol (PEG). PEG is a well-known osmotic stress inducer (Zlobin et al., 2018) and is used as a selection marker for drought stress resistance plant growth-promoting microbes (Ashry et al., 2022). PEG induced stress tolerance property of *D. lacustris* strain NSC, indicating its potential to support plant growth even under drought conditions. ACC deaminase activity further strengthens its utility as a stress-alleviating PGPR (Glick, 2014). These properties are essential for any biofertilizer strain and could enhance nutrient assimilation to improve host growth (Rao et al., 2020). Collectively, these features position *D. lacustris* NSC as a promising biofertilizer for sustainable wheat production (Brambilla et al., 2022).

Although these traits are promising, they are based primarily on the strain’s intrinsic properties. Therefore, the present study also evaluated the actual impact of *D. lacustris* NSC on wheat seed germination, growth, and yield.

Wheat seed germination is a critical determinant of crop productivity (Reed et al., 2022). Phytopathogens such as *R. solani* and *F. oxysporum* negatively impact germination and reduce yield (Agha et al., 2023). Microbes with antifungal activity against these pathogens can serve as effective biocontrol agents (Reed et al., 2022; Agha et al., 2023). Genomic evidence of antifungal genes in isolate NSC, along with experimental results, confirmed its ability to enhance wheat seed germination both under normal and pathogen-challenged conditions. Additionally, pre-inoculation with isolate NSC significantly increased seed alpha-amylase activity, essential for mobilizing stored starch into sugars to support early seedling vigor (Kumar et al., 2023). This enzymatic activity may explain the improved germination rates and seedling establishment. Pre-treatment with NSC also significantly improved shoot and root lengths in wheat seedlings under both non-stress and stress conditions. Notably, the isolate remained effective under salt stress (up to 1 M NaCl), consistent with the genomic identification of stress-resistance genes. It underlines its potential application in saline-affected soils (Zhao et al., 2016). These results highlight the dual biofertilizer and biocontrol capabilities of *D. lacustris* NSC under varied environmental conditions.

Field evaluations further validated these findings. Across multiple wheat growth stages (Feeks 1–10.5), NSC-treated plants displayed higher rhizospheric nutrient activity, including increased levels of reducing sugars, total sugars, nitrate reductase, and alkaline phosphatase activity. These enhancements indicate improved microbial-driven nutrient cycling, benefiting plant nutrient uptake and metabolism (Richardson et al., 2009). Agronomic parameters such as tiller number, spikelet density, grain weight, and overall yield significantly improved in NSC-treated plants, highlighting its potential to boost vegetative growth and reproductive output. In summary, the genomic and functional characteristics of *D. lacustris* NSC demonstrate its potential as both a plant growth-promoting biofertilizer and biocontrol agent. Its resilience to environmental stressors and ability to enhance wheat growth and yield underscore its suitability for sustainable agriculture, particularly in marginal or degraded soils with reduced chemical input.

## Data Availability

The 16S rRNA gene sequence datasets generated in this study were deposited at NCBI, PRJNA1223623 (https://www.ncbi.nlm.nih.gov/sra/PRJNA1223623).

## Footnotes

^1^ https://rast.nmpdr.org/rast.cgi
