# Supplementary material for: Assessment of the wheat growth-promoting potential of Delftia lacustris strain NSC through genomic and physiological characterization
Source: Front Microbiol. 2025 Jun 16;16:1576536. doi: 10.3389/fmicb.2025.1576536 (PMC12206771; doi:10.3389/fmicb.2025.1576536)
Supplement: Supplementary file 1 [file Data_Sheet_1.pdf]

Assessing the wheat growth promotion potential of *Delftia lacustris* strain NSC through genomic and Physiological characterization

Pinki Sharma[1], Rajesh Pandey[2,3*], Nar Singh Chauhan[1*]

[1]Department of Biochemistry, Maharshi Dayanand University, Rohtak, Haryana, India

[2]INtegrative GENomics of HOst-PathogEn (INGEN-HOPE) laboratory, CSIR-Institute of Genomics and Integrative Biology (CSIR-IGIB), Mall Road, Delhi-110007, India.

[3]Academy of Scientific and Innovative Research (AcSIR), Ghaziabad-201002, India.

*Corresponding author

Nar Singh Chauhan (nschauhan@mdurohtak.ac.in)

Rajesh Pandey (rajeshp@igib.res.in)

Running Title: *Delftia lacustris as* wheat biofertilizer.

Number of Words: 7986

Number of Figures: 4

Number of Tables: 2

Supplementary Materials

Supplementary Material SMI: Isolation of wheat rhizosphere microbes (WRMs)

Rhizospheric soil was collected from wheat plants cultivated in an experimental field at Maharshi Dayanand University, Rohtak (28° 52’ 44’’ N, 76° 37’ 19’’ E), Haryana, India. A 5.0 g soil sample was suspended in 20 mL of ultrapure sterile water for physicochemical analysis. The soil suspension was then serially diluted to 10^{-8}, and 0.1 mL of each dilution was plated onto a self-formulated minimal medium A (pH 7.2) containing urea (200 mg), calcium phosphate (250 mg), ferrous sulfate (20 mg), synthetic sea salt (200 mg), pectin (50 mg), inulin (50 mg), starch (50 mg), sorbitol (50 mg), carboxymethyl cellulose (50 mg), and ammonium sulfate (50 mg) dissolved in 100 mL of distilled water. The culture plates (in triplicate) were incubated at 16°C, 25°C, and 37°C to isolate various microbial species. Bacterial growth was monitored for 48 hours, and morphologically distinct colonies were subcultured on Luria-Bertani (LB) agar plates at their respective isolation temperature. Each morphologically diverse microbial colony was inoculated in sterile 5 mL Luria-Bertani (LB) broth and grown for 24 hours with constant shaking at 200 rpm. Genomic DNA was isolated from each microbe using the genomic DNA isolation kit (Himedia-HiPurA® Bacterial Genomic DNA Purification Kit). Primers specific to the 16S rRNA gene, like 27Fwd 5'-TCGTCGGCAGCGTCAGATGTGTATAAGAGACAGCCTACGGCWGCAG-3' and 1492Rev 5'-GTCTCGTGGGCTCGGAGATGTGTATAAGAGACAGGACTCHVG GTATCTAATCC-3', were employed to amplify the target gene. The amplification of the SSU rRNA (16S rRNA) gene was performed under optimized polymerase chain reaction (PCR) conditions using a thermal cycler (PEQ Lab, Germany). First, a denaturation step was performed at 94°C for 5 min. This was followed by 30 amplification cycles, which included denaturation at 94°C for 30 seconds, primer annealing at 58°C for 1 min, and extension at 72°C for 1.5 min. The final extension stage lasted 10 min at 72°C, as described by Gupta et al. (2016). The PCR reaction was prepared in a total volume of 25 µL, which included 12.5 µL of Q5 High-Fidelity 2X Master Mix (New England Biolabs, USA), 1 µL each of forward and reverse primers (10 µM) specific for the 16S rRNA gene, 0.5 µL of metagenomic DNA (50 ng/µL), and 10 µL of nuclease-free water. The amplified product’s quality was assessed using agarose gel electrophoresis. The amplified product was sequenced with Sanger Dideoxy chain termination sequencing methodology through the hiring services. The obtained 16S rRNA gene sequences were searched for their homologs in NCBI database through nucleotide BLAST following default parameters. Only microbial isolate

NSC shared homology with *Delftia lacustris* and was used for further characterization, while other microbial isolates were processed for downstream studies.

Supplementary Material SM2: Growth pattern analysis of microbial isolate NSC

The overnight-grown culture of *Delftia lacustris* NSC was diluted to an OD of 0.01 at 600 nm in 20 ml autoclaved LB broth and incubated at 37°C with continuous shaking. The experiment was initiated by inoculating the culture in three separate flasks containing 20 mL autoclaved LB broth, while an additional flask containing sterile 20 mL LB broth was used as a negative control. Growth was monitored by measuring absorbance at 600nm at an interval of 4 hours until the decline phase. Serial dilutions (10^{-1} to 10^{-6}) were spread on LB agar plates and incubated at 37°C for 24 hours. Colony morphology was compared to verify purity, with a negative control included to detect contamination. All experiments were repeated twice to ensure data reproducibility.

Supplementary Material SM3: Substrate utilisation assay

The substrate utilization profile of the microbial isolate NSC was evaluated using the Hi-Carbo kit (Himedia, KB009A-1KT, KB009B-1KT, and KB009C-1KT). A 50 µl aliquot of the 1.0 O.D. culture was added to Kit A, B, and C, each containing different carbohydrates to assess the isolate's ability to utilize them. Overall, three strips of each Kit A, B & C (total 9) were inoculated with 50 µl microbial culture to have three technical replicates. This experiment was conducted twice with a similar number of replicates to ensure experimental reproducibility.

Supplementary Material SM4: Amylase plate assay

The plate assay was performed utilizing agar plates supplemented with starch, composed of 2% starch and 1.5% agar. Upon solidification of the agar, a patch was placed at the centre and then incubated overnight at 37°C. A total of three plates were used to develop technical replicates. After incubation, a 1% iodine solution was applied to the agar surface, and the presence of a hydrolytic zone around the microbial growth was assessed (Swain et al., 2006). This experiment was repeated twice to check experimental reproducibility.

Supplementary Material SM5: Catalase assay

The catalase activity was assessed using the bubble test, which involves the addition of hydrogen peroxide (H_2O_2) to a bacterial culture. A small amount of the bacterial colony or culture was placed in a test tube or on a glass slide, and a few drops of 3% hydrogen peroxide were added. If catalase is present in the microbial cells, the enzyme catalase will catalyze the breakdown of hydrogen peroxide into water and oxygen. This reaction produces visible effervescence, or the formation of

bubbles, indicating a positive result. The absence of bubble formation suggests a negative result (Iwase et al., 2013). This experiment was performed with technical and biological triplicates (a total of 9 media plates were used).

Supplementary Material SM6: Pectinase assay

Microbial isolate NSC was grown on three pectinase screening agar medium (PSAM) plates. The microbial isolate NSC was streaked onto the surface of the solidified media and incubated at 30°C for a period ranging from 24 hours. After the incubation period, all three plates were flooded with a 50 mM potassium iodide-iodine solution. The presence of a clear halo zone around the colonies indicated the isolate's ability to produce pectinase (Oumer and Abate, 2018). All this experiment was performed with technical triplicates and biological duplicates (a total of 6 media plates were used).

Supplementary Material SM7: Cellulase assay

The overnight-grown culture was spot-plated onto three carboxymethylcellulose (CMC) agar, which contained 0.2% NaNO3, 0.1% K2HPO4, 0.05% MgSO4, 0.05% KCl, 0.2% (CMC) sodium salt, 0.02% peptone, and 1.5% agar. The plates were then incubated at 37°C for 48 hours. After the incubation period, the plates were flooded with a 1% hexadecyltrimethyl ammonium bromide (HAB) solution and left for a few minutes (Kasana et al., 2008). All this experiment was performed with technical triplicates and biological duplicates (a total of 6 media plates were used).

Supplementary Material SM8: Esterase activity

The overnight-grown culture was spot-plated onto three basal medium plates containing 0.5% (w/v) peptone, 0.3% (w/v) yeast extract, and 1.5% bacteriological agar (pH 7) supplemented with 1% tributyrin. The plates were then incubated at 37°C for 48 hours. After the incubation period, halozone was observed (Ramnath et al., 2017). All this experiment was performed with technical triplicates and biological duplicates (a total of 6 media plates were used).

Supplementary Material SM9: Protease activity

Microbial isolate was screened for protease production using 1% skim milk agar, prepared by dissolving 2.8 g skim milk powder, 500 mg casein enzymatic hydrolysates, 250 mg yeast extract, 100 mg dextrose, and 1.5 g agar in 100 ml distilled water, with the pH adjusted to 8.5. The isolates were spot inoculated onto three screening medium plates and incubated overnight at room temperature. The presence of a clear zone around the microbial growth was observed

(Vijayaraghavan et al., 2017). All this experiment was performed with technical triplicates and biological duplicates (a total of 6 media plates were used).

Supplementary Material SM10: Assessment of salt stress resistance physiology

The resistance of wheat rhizosphere isolates to salt was evaluated by subjecting them to varying concentrations of sodium chloride, potassium chloride, and lithium chloride. Microbial cultures of 1.0 O.D. were then inoculated into 5 ml LB broth (pH 7.0) tubes supplemented with different concentrations of NaCl (ranging from 0 to 1M w/v), KCl (ranging from 0 to 1M w/v), and LiCl (ranging from 0 to 1M w/v) separately. All these experiments were carried out with three sets of culture medium for each salt and each concentration. The tubes were incubated for 24 hours at 37°C with constant shaking at 200 rpm. Growth was assessed by measuring absorbance at 600nm after 24 hours. The resistance of wheat rhizosphere isolates to metals/metalloid was evaluated by subjecting them to varying concentrations of sodium arsenate, sodium arsenite, and cadmium chloride. Microbial cultures of 1.0 O.D. were then inoculated into 5 mL LB broth (pH 7.0) media by supplementing the medium with different concentrations of Na3AsO4 (ranging from 0 to 1000 PPM w/v), NaAsO2 (ranging from 0 to 1000 PPM w/v), and CdCl2 (ranging from 0 to 6 mM w/v). All these experiments were carried out with three sets of culture medium for each salt and each concentration. Incubated the tubes for 24 hours at 37°C with constant shaking at 200 rpm. Growth was assessed by measuring absorbance at 600nm after 24 hours.

Supplementary Material SM11: Genome Characterisation and Comparative Genomics

Genome sequencing was performed on IlluminaMiSeq using Nextera XT DNA Library Prep kit (Nextera XT DNA Sample Preparation Kit (illumina.com)). Raw reads were quality checked using FASTQC v0.11.9 (http://www.bioinformatics.babraham.ac.uk/projects/fastqc) and fastQ Validator v0.1.1 (https://github.com/statgen/fastQValidator). Contaminated reads were removed to get the corrected reads. The SPAdes v3.15.1 assembler was used for the de-novo assembly, which uses an automatic k-mer optimization approach and, thereby, a good tool for bacterial genome assembly. It uses BayesHammer to perform read error correction on each data set and Mismatch Corrector - a post processing tool, to reduce the number of mismatches in assembly using BWA tool. Further, BUSCO v5.0.0 assessment tools were used with the latest bacterial orthologue catalog (bacteria_odb10) for analyzing the completeness of a set of predicted genes in bacterial genome assemblies. BUSCO attempts to provide a quantitative assessment of the completeness in terms of the expected gene content of a genome assembly or annotated gene set.

Assembled contigs were used for functional annotation via PROKKA, and to identify the species of the organism, the SSU rRNA gene was extracted, and the BLASTn was performed. The bacteria that showed more than 97% similarity to query 16S rRNA gene sequences (Wheat rhizosphere bacteria 16S rRNA genes) were chosen for comparative analysis. Genomes were downloaded from the NCBI web server and annotated via PROKKA (doi.org/10.1093/bioinformatics/btu153). J-species software (http://jspecies.ribohost.com/jspeciesws/) was used to assess the genome level similarity using average nucleotide identity and tetra-correlation values. CRISPR/Casin, the genome was identified using the CRISPR identifier. Antibiotic resistance genes using CARD identifier where assembled contigs were used to draw a circular genomic map via Proksee tool (https://proksee.ca/). The protein features responsible for antibiotic resistance, metal/metalloid resistance, and oxidative stress resistance were identified using rapid annotation using subsystem technology (RAST) server (https://rast.nmpdr.org/rast.cgi?page=Jobs). The genome was checked for pathogenesis with the Island Viewer 4 with the default parameters (Islandviewer 4 - Genomic Island Prediction and Genome Visualization Tool (sfu.ca)). Phylogenome characterization of wheat rhizosphere microbes and shortlisted strains were plotted using roary_plots.py v0.1.0 (https://github.com/sanger-pathogens/Roary/blob/master/contrib/roary_plots/roary_plots.py). The core multiple sequence alignments was used for the inference of the phylogenomic tree using FastTree v2.1.10 (doi.org/10.1371/journal.pone.0009490).

Supplementary Material SM12: Assessment of alkaline and acid phosphatase activity

A single microbial colony was inoculated into 5 ml (in triplicate) of sterile National Botanical Research Institute's phosphate growth medium (NBRIP) (pH 7.0) and kept at 37°C with constant shaking at 200 rpm until reaching a microbial growth of 1.0 OD (600nm). Intracellular phosphate-solubilizing activity was assessed in the microbial pellets collected after centrifuging the culture at 10,000 rpm. After suspending the microbial pellet in 100 mM Tris-Cl buffer (pH 8.0), the supernatant was transferred into a microcentrifuge tube devoid of nuclease. Microbial cells were lysed by sonicating for 10 min with a cycle of 10 seconds on and 5 seconds off. A 0.3 ml microbial lysate was combined with 200µl of 100mM Tris-Cl buffer (pH 10.4) and 100µl of 4-Nitrophenyl phosphate (10mM) to measure alkaline phosphatase activity. Acid phosphatase activity was assessed by combining 0.3 ml of microbial lysate with 200 µl sodium acetate buffer (pH 4.0) and 100 µl of 4-nitrophenyl phosphate (10 mM). The enzyme assays were incubated at 37°C for 30 min, followed by the addition of 400 µl of 0.5 M NaOH solution. Absorbance was read at 405 nm,

with Tris-Cl and NaOH used as blanks. Negative enzyme activity assays were performed by replacing the buffer with lysate in the enzyme assay. Extracellular phosphate-solubilizing activity was assessed in the supernatant obtained after centrifuging an overnight-grown microbial culture at 10,000 rpm. Acid and alkaline phosphate activity was assessed with 0.3 ml of supernatant in the above-mentioned enzyme assays (Behera et al., 2017). A standard plot for 4-Nitrophenol was developed for calculating phosphatase assay using different concentrations of p-nitrophenol in the buffer.

Supplementary Material SM13: Assessment of nitrate reductase activity

A single microbial colony was inoculated in 5 ml of sterile LB broth (pH 7.0) supplemented with 0.1% potassium nitrate and incubated at 37°C with shaking (200 rpm) until reaching 1.0 OD (600 nm). Overall, three sets of tubes were inoculated with microbial colonies, and nitrite production in root extracts was measured using the method of Streeter and Devine (1983), by adding NED (0.02% in ethanol) and sulphanilamide (1% in 1.5N HCl). The resulting pink color was quantified by absorbance at 540 nm (Kim and Seo, 2018). Nitrite concentrations were determined from a sodium nitrite standard curve (R = 0.99827). All assays were performed in triplicate.

Supplementary Material SM14: Assessment of Ammonia production

Ammonia production by microbial isolate NSC was assessed by culturing it in 4% peptone broth (in triplicate) and incubating at 30 °C for seven days. After incubation, 0.5 ml of Nessler's reagent was added to the bacterial culture. The appearance of a brown to yellow coloration indicated the presence of ammonia (Bhattacharyya et al., 2020). All experiments were repeated thrice.

Supplementary Material SM15: Assessment of Siderophore Production

A single colony of the microbial isolate NSC was patched onto CAS agar plates (03) to assess siderophore production, following the method described by Himpsl and Mobley (2019). Plates were incubated at 37°C for 24 hours, and halozone was observed. This experiment was repeated thrice.

Supplementary Material SM16: Assessment of auxin production activity of wheat rhizosphere isolates

IAA production by the microbial isolate was assessed using the Salkowski reagent method. Bacterial cultures were grown in sterile LB broth (pH 7.0) with 0.1% L-Tryptophan for 5 days at 37°C with shaking (200 rpm) in the dark in three sets. After incubation, 1000 µl of Salkowski reagent (2% $FeCl_3$ in 35% $HClO_4$) was added to 1000 µl of microbial supernatant, and the color

change from brown to pink was observed after 30 minutes at room temperature (Ehmann, 1977). Absorbance was measured at 530 nm, and IAA concentrations were determined using an IAA standard curve (R = 0.9978). This experiment was repeated thrice.

Supplementary Material SM17: Assessment of drought stress tolerance ability of wheat rhizosphere isolates

Polyethylene glycol (PEG) was used as a drought stressor. Wheat rhizosphere microbe was grown at 37°C for 24 hours with continuous shaking in 5 ml sterile nutrient broth (NB) (pH 7.0). A 100ul of Microbial culture was incubated with a diverse range of PEG (0, 5%, 10%, 20%, 30%, and 40%) for 24 hours at 37°C with continuous shaking at 200 rpm. Overall, the experiment was performed in three sets, meaning three media containing tubes for each concentration. Microbial growth was observed by reading the absorbance of the culture at 600 nm using a UV-visible spectrophotometer. This experiment was repeated thrice.

Supplementary Material SM18: Assessment of 1-aminocyclopropane-1-carboxylic acid (ACC) deaminase activity of

Microbial cells were initially induced in minimal media (three sets) described by Dworkin and Foster, 1958 (doi: 10.1128/jb.75.5.592-603.1958). Harvested the cells by centrifugation at 16,000g for 5 minutes. Subsequently, the cells were washed with 0.1 M Tris-HCl (pH 7.6) and resuspended in 600 µL of 0.1 M Tris-HCl (pH 8.5). After washing, 30 µL of toluene was added to disrupt the cells, followed by vortexing for 30 seconds. Two hundred microliters of the toluenized cell suspension were mixed with 20 µL of 0.5 M ACC solution and incubated at 30°C for 15 minutes. Following the incubation, 1 mL of 0.56N HCl was added, and cell debris was removed after centrifugation of the mixture at 16,000 rpm for 5 minutes. One mL of the culture supernatant was combined with 800µL of HCl (0.56 N) and freshly prepared 300µL of DNPH reagent (0.1 g 2,4-dinitrophenyl hydrazine in 100 mL of 2 N HCl), Mixture was mixed by vortexing and incubated at 30°C for 30 minutes. After the incubation, 2 mL of NaOH (2 N) was added. The absorbance of the reaction mixture was measured at 540nm using a UV-visible spectrophotometer. ACC deaminase activity was calculated after calculating the amount of α-Ketoglutarate in the reaction mixture using a standard curve (R=0.9998) generated using different concentrations of α-Ketoglutarate. This experiment was repeated thrice.

Supplementary Material SM19: Assessment of *Delftia lacustris strain* NSC biofertilizer and biocontrol properties in laboratory conditions

The biocontrol potential was evaluated against *Rhizoctonia solani*, and *Fusarium oxysporum*, soil-borne pathogens affecting the crop globally. *Rhizoctonia solani* causes root rot and damping-off, leading to poor seedling establishment and yield loss. At the same time, *Fusarium oxysporum* is responsible for Fusarium wilt, which disrupts water and nutrient flow in the plant. The assessment focused on their effects on seed germination efficiency and wheat seedlings' root and shoot lengths (Sharma et al., 2024a; Singh and Kayastha, 2014). To investigate the role of *Delftia lacustris* strain NSC in seed germination under saline conditions, seeds were soaked in an overnight-grown microbial culture (cell density of 10^{11} cells/mL), supplemented with NaCl concentrations ranging from 0 to 1 M, for 16 hours at 37°C. Control seeds were soaked directly in NaCl solutions of equivalent concentrations for 16 hours at 37°C. Following the soaking treatment, seeds were wrapped in germination sheets and placed in 50 mL culture tubes containing 5 mL Hoagland solution. The tubes were incubated in the dark at ambient temperature (25°C) for 7 days. After incubation, wheat seed germination percentage, alpha-amylase activity, and root and shoot lengths were measured (Sharma et al., 2024a; Singh and Kayastha, 2014).

Supplementary Material SM20: Assessment of *Delftia lacustris strain* NSC biofertilizer properties under field conditions: Wheat cultivar 306 (WC-306) plants were cultivated in an experimental field in the botanical garden at Maharshi Dayanand University, Rohtak (28° 52 44'' NL and 76° 37 19'' EL), Haryana, India. Overall, the experiment was carried out as per the experimental layout.

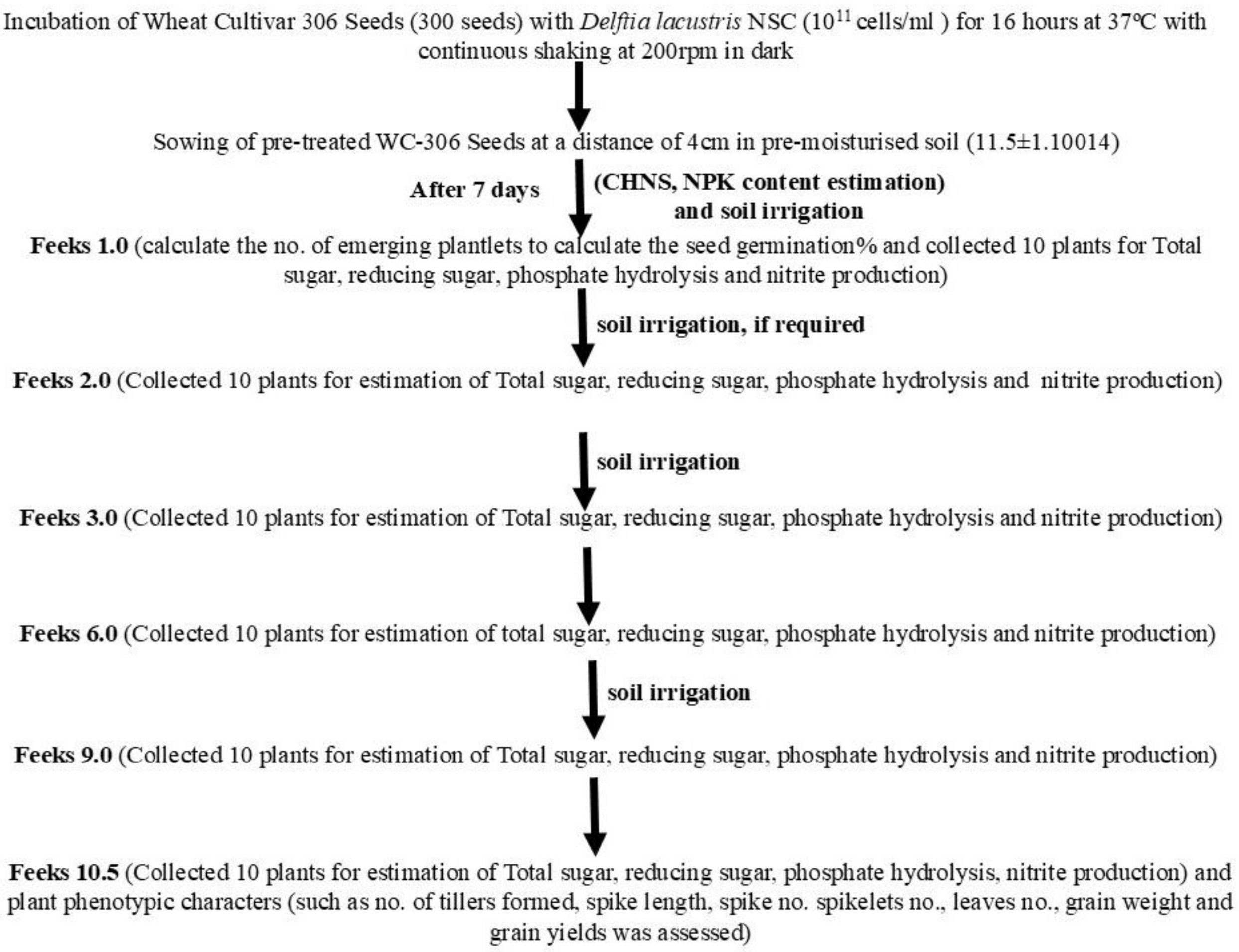

Experimental layout to assess biofertilizer properties *Delftia lacustris strain* NSC under field conditions

Wheat cultivar 306 (WC-306) plants were cultivated in an experimental field in the botanical garden at Maharshi Dayanand University, Rohtak (28° 52 44’’ NL and 76° 37 19’’ EL), Haryana, India. The WC-306 is a high-yielding cultivar suited for the rabi season in India. For optimal growth, 4 to 6 irrigations are required at critical growth stages such as emergence, tillering, internode elongation, and flowering, depending on soil type and climatic conditions. Wheat seeds were harvested at various Feeks (1.0, 2.0, 3.0, 6.0, 9.0, and 10.5 (https://www.sunflower.k-state.edu/agronomy/wheat/wheatdevelopment.html)). Wheat rhizosphere samples were processed immediately after collection. Roots of WC-306 plants treated with *Delftia lacustris* strain NSC were analyzed for their phosphate-solubilizing ability (Behera et al., 2017), nitrate reductase activity (Kim and Seo, 2018), total sugar content (Ludwig and Goldberg, 1956), and reducing sugar content (Khatri and Chhetri, 2020), with comparisons made to untreated WC-306 plants.

Additionally, various growth and yield parameters were assessed in both treated and untreated plants, including seed germination percentage, the number of tillers per plant, number of leaves per plant, number of spikes per plant, spike length, number of spikelets per spike, and grain yield, to evaluate the effects of *Delftia lacustris* strain NSC on plant growth and productivity.

Supplementary Material SM21: Estimation of total sugar content

WC-306 plant roots treated with *Delftia lacustris* NSC were collected at various weeks (1.0 to 10.5) and analyzed for total sugar content using the Anthrone method by Ludwig and Goldberg (1956). Roots (100 mg) were crushed in 1 ml ultrapure water, mixed with 4 ml Anthrone reagent, and heated at 99°C for 5 minutes. After cooling, absorbance was measured at 625 nm, with water and Anthrone as the control. A dextrose standard curve was used for quantification.

Supplementary Material SM22: Estimation of reducing sugar content

WC-306 plant roots treated with *Delftia lacustris* NSC were harvested at various weeks (1.0 to 10.5) and analyzed for reducing sugar content using the DNS method (Khatri and Chhetri, 2020). Roots (100 mg) were crushed in 1 ml ultrapure water, mixed with 1 ml DNS reagent, and heated at 99°C for 5 minutes. After cooling, absorbance was measured at 540 nm, with water and DNS as the control. Reducing sugar content was quantified using a dextrose standard curve, and all assays were performed in technical triplicate.

Supplementary Material SM23: Estimation of nitrite production

Plant roots treated with *Delftia lacustris* NSC were collected at different weeks (1.0 to 10.5) and assessed for nitrate reductase activity using the method by Kim and Seo (2018). Roots (100 mg) were homogenized in 1 ml PBS buffer (pH 7.0), and 1 ml of the extract was mixed with reagents containing sulfanilamide and N-(1-naphthyl)ethylenediamine. Absorbance was measured at 550 nm, with a blank lacking root extract. Nitrite concentration was determined using a standard curve (R = 0.99827), and all assays were performed in technical triplicate.

Supplementary Material SM24: Estimation of phosphate hydrolysis

Roots treated with *Delftia lacustris* NSC were collected at various growth stages (1–10.5 feeks) and analyzed for phosphate hydrolysis activity using the method of Streeter and Devine (1983). For each assay, 100 mg of wheat roots were homogenized in 1 ml of buffer, and alkaline or acid phosphatase activity was measured using 4-nitrophenyl phosphate as the substrate. Reactions were incubated at 37°C for 30 minutes, stopped with NaOH, and absorbance was read at 405 nm. A

standard curve of p-nitrophenol was used for quantification, and all assays were conducted in triplicate.

Supplementary Material SM25: Alpha-amylase activity assessment of microbial isolate NSC

Alpha amylase activity of WC-306 seeds treated with *Delftia lacustris* NSC was assessed. A 100mg of wheat rhizosphere samples were homogenized in 1ml PBS buffer pH 7.0 using a tissue homogenizer for 5 minutes at room temperature. The α-amylase hydrolytic activity was routinely evaluated according to the protocol outlined by Kayastha et al., 2007. A mixture comprising 500µl of 1% starch soluble (w/v) with 300µl of 100mM sodium acetate buffer (pH 5.0), and 100 µl of ultrapure water incubated at 68°C for 10 minutes. The enzymatic reaction was initiated with the addition of 100µl of homogenate. After 5 minutes, the reaction was terminated by the addition of 500µl of 1 N HCl, followed by rapid cooling. A200µl of resulted reaction mixture was combined with 100µl of 1 N HCl, and 100µl of iodine solution. The total volume was adjusted to 15mL, and absorbance was recorded at 610 nm using a UV-visible spectrophotometer. One enzyme unit of α-amylase activity was defined as the enzyme amount causing a decrease in absorbance of 0.05 in the starch-iodine color complex under the assay conditions. All assays were performed in technical triplicate.

Supplementary Material SM26: Plant phenotypic characters assessment of microbial isolate NSC

This study evaluated the effect of *Delftia lacustris* strain NSC on wheat growth and productivity by comparing key growth and yield parameters between treated and untreated plants. Parameters included seed germination, number of tillers, leaves, and spikes per plant, spike length, number of spikelets per spike, grain weight and grain yield -all indicators of plant health and productivity.

Supplementary Table S1: Substrate utilization profile of *Delftia lacustris* strain NSC with other phylogenetic-related *Delftia* species

Sr. No.	Substrate	*Delftia lacustris* NSC	*Delftia lacustris* LzhVag01	*Delftia tsuruhatensis* CM13	*Delftia tsuruhatensis* NBRC 16741	*Delftia acidovorans*
1	Lactose	+	+	ND	ND	ND
2	Xylose	+	ND	ND	ND	ND
3	Maltose	+	ND	ND	ND	+
4	Fructose	ND	+	+	ND	ND
5	Dextrose	+	+	+	ND	+
7	Raffinose	+	ND	ND	ND	ND
8	Trehalose	+	+	ND	ND	ND
9	Melibiose	ND	ND	ND	ND	ND
10	Sucrose	+	+	ND	ND	ND
11	L-Arabinose	ND	ND	ND	ND	+
12	Mannose	ND	ND	+	ND	+
13	Inulin	+	+	ND	ND	ND
14	Glycerol	ND	+	ND	ND	ND
15	Dulcitol	+	ND	ND	ND	ND
16	Mannitol	+	+	ND	ND	+
17	Adonitol	+	ND	ND	ND	ND
18	Rhamnose	+	ND	ND	ND	ND
19	Xylitol	+	ND	+	ND	ND
20	ONPG	+	+	ND	ND	ND
21	Esculin hydrolysis	ND	ND	ND	+	ND
22	D-Arabinose	ND	ND	ND	ND	ND
23	Citrate utilization	ND	ND	ND	+	ND
24	Malonate utilisation	ND	ND	ND	ND	ND
25	Sorbose	+	+	ND	ND	+

Here ND: Not defined in the literature.

Supplementary Table S2: Comparative analysis of antibiotic susceptibility profile of *Delftia lacustris* NSC with other phylogenetic similar *Delftia* strains.

**Sr. No.**	**Antibiotic**	***Delftia lacustris* NSC**	***Delftia lacustris* LzhVag01**	***Delftia tsuruhatensis* CM13**	***Delftia tsuruhatensis* NBRC 16741**	***Delftia acidovorans***
1	Amikacin	ND	ND	+	+	ND
2	Amoxicillin	ND	ND	ND	+	ND
3	Bacitracin	+	+	ND	ND	ND
4	Cephalothin	ND	ND	ND	ND	ND
5	Erythromycin	ND	+	ND	+	ND
6	Novobiocin	+	+	ND	ND	ND
7	Oxytetracyclin	+	ND	ND	ND	
8	Vancomycin	+	+	ND	ND	ND
9	Ceflnaxone	ND	ND	+	ND	ND
10	Ceftazidime	+	+	ND	ND	+
11	Cefotaxime	ND	ND	+	ND	ND
12	Lincomycin	ND	+	ND	ND	ND
13	Netillin	ND	ND	+	ND	ND
14	Ofloxacin	+	ND	ND	ND	ND

Here, ND is not defined in the literature.

Supplementary Table S3: A Comparison of the growth parameters of *Delftia lacustris* NSC with other *Delftia* strains

Sr no.	Microbes	pH	Temperature (˚C)	Minimum Inhibitory Concentration						
				**NaCl**	**KCl**	**LiCl**	**As(III)**	**As(V)**	**$CdCl_2$**	**H_2O_2**
1	***Delftia lacustris* NSC**	5-10	20-55	1750mM	2000 mM	1750 mM	1500 PPM	1000 PPM	6mM	12.5mM
2	***Delftia lacustris* LzhVag01**	6-9	15-30°C	102.7mM	ND	ND mM	ND	1000 PPM	ND	ND
3	***Delftia tsuruhatensis* CM13**	5 to 9	20 to 40	900mM	ND	ND	ND	ND	ND	ND
4	***Delftia tsuruhatensis* NBRC 16741**	5-10	20–45	1000mM	ND	ND	ND	ND	ND	ND
5	***Delftia acidovorans***	5-10	20–40	530mM	ND	ND	ND	ND	ND	ND

Here ND: Not defined in the literature.

Supplementary Table S4: Average nucleotide identity (ANI) of *Delftia lacustris* NSC with other *Delftia* species.

	**Microbial isolate NSC**	***Delftia acidovorans***	***Delftia lacustris***	***Delftia* sp hk171**	***Delftia tsuruhatensis* NBRC 16741**	***Delftia tsuruhatensis T7***
**Microbial isolate NSC**	*	94.54	**98.08**	94.65	98.05	93.16
Delftia acidovorans	94.48	*	94.59	97.4	81.45	89.92
Delftia lacustris	**98.28**	94.34	*	94.39	84.85	92.62
***Delftia* sp hk171**	94.42	97.31	94.53	*	80.95	89.51
***Delftia tsuruhatensis* NBRC 16741**	97.65	95.05	96.45	93.93	*	95.79
***Delftia tsuruhatensis* T7**	97.68	93.87	96.76	93.73	88.31	*

Supplementary Table S5: Tetra correlation among *Delftia lacustris NSC* and other *Delftia* species by a wide distribution of Z-score.

Organism	Z- Score
Delftia lacustris LMG 24775	0.99989
Delftia tsuruhatensis CM13	0.99983
Delftia tsuruhatensis NBRC 16741	0.99976
Delftia acidovorans CCUG 15835	0.99976
Delftia acidovorans CCUG 274B	0.99975
Delftia sp. 670	0.99964
Delftia sp. Cs1-4	0.99959
Delftia sp. JD2	0.99957
Delftia tsuruhatensis 391	0.99955
Delftia acidovorans SPH-1	0.99955
Delftia acidovorans FDAARGOS_997	0.9995
Delftia acidovorans 2167	0.99945
Delftia acidovorans NBRC 14950	0.99933
Delftia tsuruhatensis MTQ3	0.99925
Delftia sp. RIT313	0.99913
Comamonas terrae NBRC 106524	0.98431
Comamonas terrae TISTR 1906	0.9843
Comamonas phosphati CGMCC 1.12294	0.98044
Acidovorax sp. CF316	0.97175
*Xenophilus arseniciresisten*s YW8	0.96631
Acidovorax sp. Root217	0.96576
Acidovorax sp. Root219	0.96546
Paenacidovorax monticola KACC 19171	0.9651
Pseudorhodoferax soli DSM 21634	0.96355
[*Acidovorax*] ebreus TPSY	0.96319
Pseudorhodoferax sp. Leaf267	0.96281
Diaphorobacter nitroreducens DSM 15985	0.96259
Alicycliphilus denitrificans K601	0.96209
Acidovorax sp. JS42	0.96199
Alicycliphilus sp. B1	0.96192
Diaphorobacter sp. J5-51	0.96168
Pseudorhodoferax sp. Leaf274	0.96126
Alicycliphilus denitrificans BC	0.96087
Pseudorhodoferax sp. Leaf265	0.96075
Pseudorhodoferax aquiterrae KCTC 23314	0.9592
Acidovorax sp. MR-S7	0.95598

Comamonas aquatica NBRC 14918	0.95559
Comamonas aquatica DA1877	0.95538
Comamonas granuli NBRC 101663	0.95212
Simplicispira lacusdiani CPCC 100842	0.95101
Variovorax terrae CYS-02	0.94929
Comamonas guangdongensis CCTCC AB2011133	0.94658
Pulveribacter suum SC2-7	0.94495
Paracidovorax wautersii DSM 27981	0.9425
Paracidovorax anthurii CFPB 3232	0.94081
Paracidovorax konjaci DSM 7481	0.93916
Comamonas testosteroni TK102	0.93781
Diaphorobacter limosus Y-1	0.9371
Simplicispira sedimenti W1-6	0.93708
Acidovorax soli DSM 25157	0.93689
Aquabacterium soli SJQ9	0.93507
Comamonas testosteroni KF-1	0.93505
Acidovorax sp. Leaf191	0.93494
Acidovorax sp. Leaf76	0.93492
Acidovorax sp. Leaf84	0.93414
Curvibacter sp. PAE-UM	0.93308
Ramlibacter rhizophilus CCTCC AB2015357	0.93306
Hydrogenophaga borbori LA-38	0.93269
Comamonas testosteroni JL40	0.93219
Paracidovorax cattleyae DSM 17101	0.93216
Paenacidovorax caeni R-24608	0.93196
Paracidovorax oryzae ATCC 19882	0.93158
Paenacidovorax caeni R-24608	0.93154
Paracidovorax avenae ATCC 19860	0.93071
Comamonas endophytica 5MLIR	0.9303
Acidovorax delafieldii 2AN	0.93007
Paracidovorax valerianellae DSM 16619	0.92985
Comamonas terrigena NBRC 13299	0.92962
Comamonas terrigena NBRC 13299	0.92908
Acidovorax sp. Leaf78	0.92891
Comamonas terrigena NCTC1937	0.92888
Comamonas testosteroni WDL7	0.92886
Variovorax soli NBRC 106424	0.9287
Melaminivora suipulveris SC2-9	0.92835
Comamonas thiooxydans DS1	0.92826
Paracidovorax citrulli AAC00-1	0.92712

Bordetella hinzii 5132	0.92707
Comamonas resistens ZM22	0.92684
Comamonas thiooxydans JL14	0.92648
Comamonas antarctica 16-35-5	0.92631
Paracidovorax citrulli ICMP 7500	0.92627
Comamonas thiooxydans DF1	0.92603
Bordetella hinzii 1277	0.92599
Comamonas thiooxydans JC9	0.92593
Paracidovorax citrulli DSM 17060	0.92586
Bordetella hinzii NCTC13199	0.92582
Comamonas sp. E6	0.9258
Comamonas thiooxydans DF2	0.92573
Comamonas thiooxydans JC13	0.92573
Bordetella hinzii F582	0.9257
Comamonas thiooxydans DSM 17888	0.9257
Bordetella hinzii L60	0.92569
Bordetella hinzii LMG 13501	0.92568
Comamonas thiooxydans DSM 17888	0.92558
Comamonas thiooxydans DSM 17888	0.92558
Bordetella hinzii OH87 BAL007II	0.9255
Comamonas thiooxydans JC12	0.92546
Comamonas thiooxydans JC8	0.92536
Acidovorax sp. Root275	0.92513
